# Steric Scaling of Hydrogen Transport in FCC Transition Metals for Mechanically Consistent Molecular Dynamics Simulations

**DOI:** 10.1021/acsomega.6c05027

**Published:** 2026-10-15

**Authors:** Emmanuel Peters-Teke Tebo, Sina Karimzadeh, Nyuytifo Emmanuel Wiykiynyuy, Tien-Chien Jen

**Affiliations:** Department of Mechanical Engineering Science, 61799 University of Johannesburg, Johannesburg, Gauteng 2006, South Africa

## Abstract

Hydrogen diffusion in face-centered cubic metals is examined using replicate-resolved molecular dynamics that quantifies temperature-dependent transport and lattice stability. Transport in Ni, Co and Pd is modeled with a hybrid embedded-atom method/Lennard-Jones framework at 800, 900, 1000, and 1250 K. Rather than fitting hydrogen–metal interactions to a single diffusivity, migration energetics are constrained through steric scaling of the FCC interstitial geometry. All baseline finite-concentration parametrizations remain structurally stable under the monitored lattice-volume criteria. Volume fluctuations stay below 0.15% and swelling below 0.09%. Late-time mean-squared displacement remains linear in all accepted trajectories. Four-temperature Arrhenius fits are stable for Ni, Co, and uniform Pd, while scaled Pd remains less constrained. The expected diffusivity hierarchy *D*_Pd_ > *D*_Co_ ≈ *D*_Ni_ is recovered. Steric scaling lowers Pd diffusivity at every temperature while leaving Ni and Co unchanged, since their interaction parameters are fixed. Pathway-resolved single-H calculations give 0 K barriers of 0.1516 eV for Ni and 0.1294 eV for Co. For Pd, scaling raises the verified static barrier from 0.0234 to 0.0398 eV. A higher-loading Pd test retains the diffusivity reduction while lattice swelling remains bounded. Steric scaling therefore modulates the resolved migration landscape while preserving lattice stability and the observed finite-temperature diffusivity trends.

## Graphical abstract

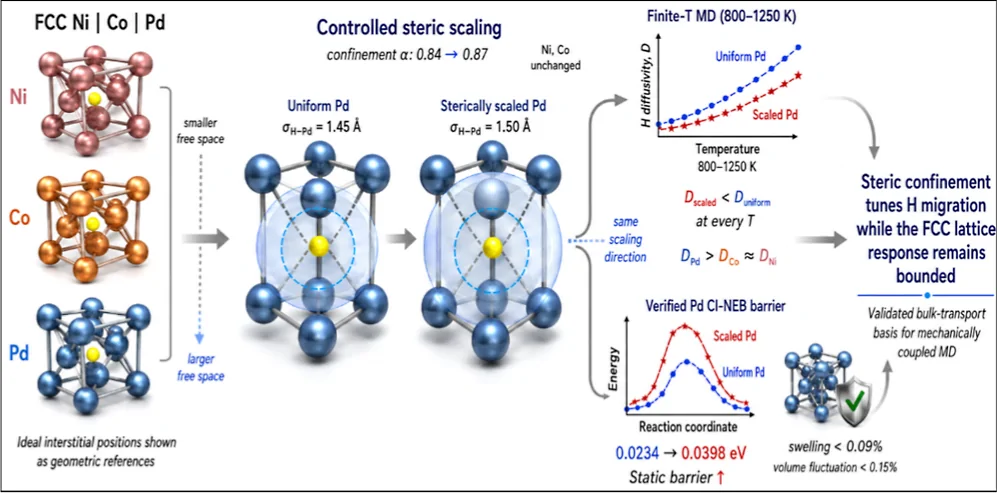

## 1 Introduction

Hydrogen transport governs the onset and progression of hydrogen-assisted degradation in structural metals.^1–5^ In face-centered cubic (FCC) lattices, hydrogen transport proceeds through thermally activated transitions within an interstitial network governed by lattice geometry and local site stability.^6–9^ Whether degradation proceeds through hydrogen-enhanced localized plasticity, decohesion, or stress-assisted diffusion, hydrogen must first redistribute within the lattice in response to evolving stress fields and microstructural features.^10–14^ When this redistribution is not accurately captured, apparent stress-hydrogen coupling in atomistic simulations may reflect numerical artifacts rather than physical mechanisms.^1,9,15–17^ Local structural defects further modulate hydrogen transport paths, as shown for stainless-steel passivation films.^18^ Experimental studies report higher hydrogen mobility in palladium (Pd) than in nickel (Ni) or cobalt (Co), consistent with the larger interstitial free volume afforded by its lattice parameter.^6,7,19–21^

Despite this experimental basis, atomistic simulations reproduce these trends inconsistently. Large-scale molecular dynamics (MD) studies commonly adopt hybrid interaction models in which metal–metal bonding is described by embedded atom method (EAM) potentials and hydrogen–metal interactions by simplified pairwise functions.^16,22,23^ Hydrogen self-diffusivities are typically extracted from the long-time linear regime of the mean squared displacement (MSD) via the Einstein relation, with activation energies obtained from Arrhenius fits across a temperature range.^1,8,24–28^ The same MSD logic underlies related diffusion-coefficient studies, where temperature and molecular-scale descriptors influence the fitted parameters.^29,30^ In practice, however, hydrogen–metal cross-interactions are often calibrated by matching diffusion at a single temperature.^28,31^ Such single-point calibrations do not ensure physically meaningful migration energetics, Arrhenius consistency, or mechanical stability.^6,8,32^ They can also obscure transport–stress coupling in later simulations.^1,8,33–36^ A recurring modeling failure is an interstitial energy landscape that produces cage-like motion, excessive mobility, or unstable reference sites. These trajectories still yield MSD slopes but often deviate from Arrhenius behavior.^17,37^ Departures from a single Arrhenius slope can reflect mechanism changes, concentration effects, or model artifacts and therefore require explicit diagnosis.^6,8,26^

Beyond transport kinetics, hydrogen insertion produces measurable lattice expansion, often characterized through an effective partial molar volume response linking hydrogen content to volumetric dilation.^12,36,38–40^ In mechanically coupled formalisms, this expansion generates hydrostatic stress fields that influence the chemical potential driving redistribution.^12,14–44^ Treating hydrogen as sterically negligible fails to capture this coupling, while overly large interaction radii exaggerate swelling and induce structural softening. Hydrogen-induced lattice strain measurements therefore provide an independent mechanical constraint on interaction model realism.^7,40,42,45,46^ Reactive force fields and machine-learning potentials offer improved chemical fidelity for hydrogen–metal systems.^37,47^ For large-scale mechanically oriented simulations, however, they remain impractical because of computational cost and system-specific parametrization.^22,48^ When surface chemistry and reactive processes are out of scope, this added complexity does not necessarily improve mechanical interpretability. This motivates scalable nonreactive frameworks validated against crystallographic consistency, diffusive MSD behavior, Arrhenius trends, and plausible lattice dilation. Despite established descriptions of metallic bonding^49,50^ and extensive experimental diffusion data,^1,6,8,31,51^ a framework that constrains hydrogen–metal interactions using FCC interstitial geometry and validates transport through temperature-dependent behavior across multiple metals remains largely unexplored.^14,52,53^

This work introduces a steric-scaled calibration framework for hydrogen transport in FCC transition metals. Hydrogen diffusion is examined in single-crystal Ni, Co, and Pd using a hybrid EAM/Lennard-Jones interaction model over four temperatures (800, 900, 1000, and 1250 K). Rather than fitting hydrogen–metal parameters to a single experimental diffusivity, the cross-interactions are constrained through the geometric relationship between the Lennard-Jones minimum interaction distance and the octahedral nearest-neighbor distance. The framework is evaluated through two complementary validation layers. Internal numerical validation assesses structural stability, late-time MSD linearity, and replica reproducibility. Temperature-dependent validation assesses Arrhenius fit quality, regression robustness, and recovery of the expected FCC transport hierarchy. By emphasizing comparative transport trends and temperature-dependent fidelity rather than absolute diffusion magnitudes, the approach provides a physically grounded foundation for mechanically coupled MD simulations of hydrogen-assisted deformation and redistribution.

## 2 Methodology

### 2.1 Simulation Framework

All simulations were performed using LAMMPS.^54^ Hydrogen diffusion was examined in fully periodic single-crystal FCC lattices of Ni, Co, and Pd, which span distinct lattice parameters within a common FCC crystallographic reference geometry. The objective is not to reproduce absolute experimental diffusivities at a single temperature. It is to establish barrier-controlled hydrogen transport that is internally consistent across materials and suitable for mechanically coupled simulations. Hydrogen was treated as a low finite-concentration interstitial population during production MD. Surface chemistry, bond breaking, phase transformations, and stress-driven transport were excluded to isolate intrinsic bulk diffusion governed by lattice geometry and finite-temperature energetics. The baseline MD protocol and interaction model were applied uniformly across systems. Static pathway calculations were selected only after identifying the relevant local minima and end point topology.

### 2.2 Interaction Potentials

Metal–metal interactions were described using the Zhou–Johnson–Wadley EAM alloy potential,^55^ used without modification to hold baseline metallic bonding, elastic response, and thermal expansion fixed across all simulations. Hydrogen–metal cross-interactions were represented using a Lennard-Jones (12-6) potential with a cutoff of 8.0 Å, combined with the EAM description via a hybrid scheme. The LJ form was selected because its size parameter σ and well depth ε give direct, independent control over steric confinement and attractive interaction strength at interstitial sites. Because σ sets the onset of short-range repulsion, it sets the characteristic H–metal interaction length scale within the lattice. It therefore serves as a geometric control variable for modulating migration barriers without altering the metal–metal EAM description.^22,56–58^ More complex hydrogen–metal descriptions, including reactive bond-order potentials,^37,47^ and machine-learning interatomic potentials, can reproduce local chemical environments with higher fidelity. For large-scale mechanically oriented simulations where surface reactions are out of scope, this added parametrization cost does not necessarily improve the description of geometry-governed interstitial migration.^22,31,48,59^ Hybrid EAM/LJ frameworks have been employed for hydrogen in metallic lattices at system sizes relevant to mechanically coupled simulations.^22,31^

Rather than fitting hydrogen–metal interaction parameters to reproduce diffusion at a single temperature, these cross-interactions were constrained using FCC crystallographic geometry. FCC lattices contain ideal octahedral and tetrahedral interstitial positions that define the crystallographic reference network for hydrogen transport.^6,7,19,60^ The body-center octahedral site (1/2, 1/2, 1/2), tetrahedral site (1/4, 1/4, 1/4), and neighboring octahedral site (1/2, 0, 0) are shown in Figure 1c. Their local stability and transition-state roles depend on the interaction model and cannot be assigned from crystallographic geometry alone. These positions were therefore treated as geometric reference sites rather than assumed minima or saddle points. Relevant local minima and connecting minimum-energy pathways were determined explicitly using pathway analysis and CI-NEB, as described in Section 2.5.

**Figure 1:**
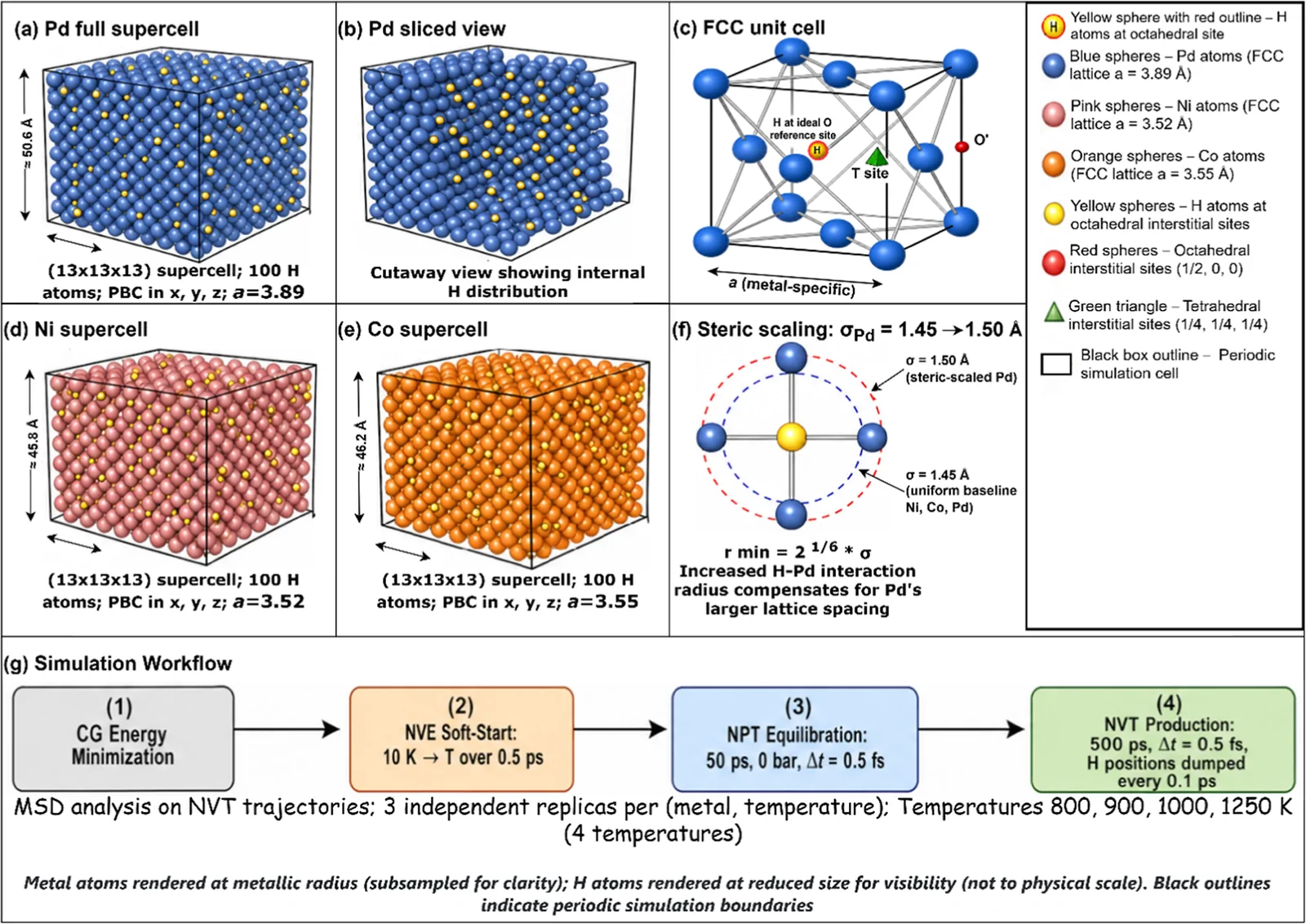
(a,b) Full and cutaway views of the Pd 13 × 13 × 13 FCC supercell (*a* = 3.89 Å; box ≈ 50.6 Å) with 100 H atoms (PBC). (c) FCC unit cell showing ideal octahedral and tetrahedral reference positions used for pathway analysis. (d,e) Equivalent Ni (*a* = 3.52 Å; box ≈ 45.8 Å) and Co (*a* = 3.55 Å; box ≈ 46.2 Å) supercells. (f) Steric scaling of H–Pd LJ radius: σ: 1.45 → 1.50 Å, *r*_min_ = 2^1/6^σ. (g) Four-stage MD protocol: conjugate-gradient minimization → *NVE* soft-start (5000 steps at 0.1 fs) → *NPT* (50 ps, 0 bar) → *NVT* production (500 ps, Δ*t* = 0.5 fs); three replicas per metal-temperature at *T* = 800, 900, 1000, 1250 K. Atom sizes not to scale.

Hydrogen–hydrogen interactions (ε = 0.001 eV, σ = 1.0 Å) were included to prevent numerical overlap while avoiding appreciable attractive H–H binding. Two parametrizations were examined under otherwise identical conditions: a uniform baseline model and a sterically scaled model. The well depth ε = 0.04 eV was held constant across metals and models. Therefore, σ was the only H–metal interaction parameter changed between the two parametrizations. In the uniform model, σ = 1.45 Å was applied to all three metals. In the sterically scaled model, σ for Pd was increased to 1.50 Å to partially compensate for its larger FCC interstitial free volume, as shown schematically in Figure 1f, while Ni and Co retained σ = 1.45 Å. Table 1 summarizes the complete parametrization. The associated Lennard-Jones minimum distances and confinement ratios are reported in Section S.1 and Table S1.

**Table 1:** Hydrogen–Metal Lennard-Jones Interaction Parameters

Metal	Model	σ (Å)	ε (eV)
Ni	Uniform/Scaled	1.45	0.04
Co	Uniform/Scaled	1.45	0.04
Pd	Uniform	1.45	0.04
Pd	Scaled	1.50	0.04

### 2.3 Computational System Definition and Initialization

Each system comprised a (13 × 13 × 13) FCC supercell (8788 metal atoms) with lattice parameters of 3.52 Å (Ni), 3.55 Å (Co), and 3.89 Å (Pd), consistent with experimental and first-principles values.^19,40^ Periodic boundary conditions were applied in all directions. One hundred hydrogen atoms were inserted randomly within a central cubic region, excluding one lattice spacing from each boundary. This corresponds to approximately 1.1 at. %, expressed as the hydrogen-to-metal atom ratio (100 H per 8788 metal atoms), the convention used for all concentrations in this work. The loading was chosen as a low finite-concentration baseline that provides sufficient hydrogen for stable ensemble-averaged MSD extraction.^20,27,44^ The 1.1 at. % condition is treated as the baseline finite-concentration MD state rather than an infinite-dilution limit. Possible concentration effects on the fitted Arrhenius barrier are therefore not excluded. A 5.0 at. % Pd sensitivity test examines whether the scaling direction persists at higher loading. The single-H static calculations isolate the 0 K migration landscape in the single-H limit. Higher concentrations can reduce diffusivity through site blocking, H–H correlations, and local strain fields and can shift the fitted effective activation energy. Related simulations of hydrogen adsorption and diffusion on metal nanoparticles report similar adsorbate–adsorbate effects under hydrogen-rich conditions.^61,62^

Three independent replicas per metal-temperature condition were generated using distinct insertion seeds (Section S.2). The temperature window of 800–1250 K was selected on phase-stability and sampling grounds. The 800 K floor keeps FCC Co in the high-temperature regime used for the comparative framework^63,64^ and provides thermally activated hopping sufficient for converged linear MSD within the 500 ps production window. The 1250 K ceiling is about 0.72 of the Ni melting point, 1728 K, and remains below the melting threshold. This limits premelting effects and preserves the intended bulk interstitial-diffusion regime. The constructed supercells for all three metals, together with the FCC unit cell interstitial site architecture and the four-stage simulation workflow, are illustrated in Figure 1.

### 2.4 Equilibration and Diffusion Sampling

Initial configurations were relaxed by conjugate gradient energy minimization (force tolerance 5 × 10^–6^ eV/Å, energy tolerance 10^–4^ eV). A stabilization stage of 5000 steps at 0.1 fs under velocity-limited integration (*NVE*/limit) with a Langevin thermostat ramping from 10 K to the target temperature was then applied to suppress large forces arising from hydrogen insertion,^3,26,65^ without imposing an artificial quench on the host lattice. The complete four-stage protocol is shown in Figure 1g.

The integration time step was subsequently set to 0.5 fs for all production stages. Lattice relaxation was performed under an *NPT* ensemble (Nosé–Hoover thermostat and barostat; temperature damping 0.1 ps, pressure damping 1.0 ps) at zero external pressure for 100,000 steps (50 ps). Volume statistics were averaged over the final 10 ps to confirm convergence to a stationary equilibrium state.^44,66^ Diffusion trajectories were then generated under an *NVT* ensemble (Nosé–Hoover thermostat, 0.1 ps damping) at the equilibrated volume for 1,000,000 steps (500 ps). Hydrogen positions with periodic image flags were recorded every 200 steps (0.1 ps). Separating *NPT* equilibration from fixed-volume *NVT* production samples diffusion at a well-defined thermodynamic state and avoids coupling between cell fluctuations and MSD slopes.^17,66^

### 2.5 Diffusion Analysis and Validation Framework

Hydrogen diffusion was quantified using mean-squared displacement (MSD) analysis under *NVT* conditions following equilibration, with only structurally stable trajectories retained. Diffusion coefficients were extracted from the linear MSD regime using the Einstein relation

1 $$D=\frac{1}{6}\;\frac{d}{dt}\left\langle r^{2}\left(t\right)\right\rangle$$

where *r* ^2^(*t*) is the ensemble-averaged MSD. A multiorigin approach was employed to improve statistical accuracy, and linear regression was performed over adaptive late-time windows satisfying *R* ^2^ ≥ 0.99, a minimum duration of 50 ps, and negligible slope variation under window refinement.

Parameterizations were evaluated using two complementary validation layers. Internal numerical validation assessed structural stability, late-time MSD linearity, and reproducibility across independent replicas. Temperature-dependent validation assessed monotonic diffusivity trends, Arrhenius regression quality, and recovery of the expected FCC transport hierarchy (Pd > Co ≈ Ni).^8,24,27^

Temperature-dependent diffusivities were analyzed using the Arrhenius relation

2 $$D=D_{0}\;\exp\left(-\frac{Q}{k_{B}T}\right)$$

where *Q* is the activation energy, *D*_0_ is the pre-exponential factor, and *k*_B_ is Boltzmann’s constant. Activation energies were obtained from linear regression of

3 $$\ln D=\ln D_{0}-\frac{Q}{k_{B}T}$$

This treatment infers an effective activation energy from the temperature dependence rather than from a single-temperature descriptor.^30^ Arrhenius robustness was audited using ordinary least-squares confidence intervals, bootstrap resampling, leave-one-temperature-out fits, and adjacent-temperature apparent slopes. These diagnostics distinguish weak four-temperature regression from a static-pathway inconsistency. They do not establish migration topology independently.

Static pathway calculations were performed only after identifying the relevant interstitial topology. Dedicated high-frequency Ni and Co trajectories tested the dynamically relevant O-site end point family. Single-H local-stability calculations then identified valid O, T, and Pd O* basins. CI-NEB calculations were constructed between verified local minima using multiple independently initialized bands. Image-count and force-convergence tests established numerical stability of the final barriers. Full trajectory, basin-mapping, pathway-initialization, and convergence details are provided in Section S.7.

## 3 Results

### 3.1 Structural Stability

Structural stability of hydrogen-containing FCC lattices was evaluated during zero-pressure *NPT* equilibration for Ni, Co, and Pd. Both the uniform and steric-scaled hydrogen–metal parametrizations were assessed using identical simulation protocols and acceptance criteria. Across all metals and temperatures, the cells converged rapidly to stationary volume fluctuations. No unbounded expansion, lattice collapse, or nonstationary drift was observed in any trajectory. Late-time volume statistics confirmed bounded oscillatory behavior.

Figure 2A,B summarize the normalized thermodynamic volume fluctuation amplitude, σ_*V*_/⟨*V*⟩, against temperature for the uniform and scaled parametrizations. The fluctuation amplitude varied with temperature but was not strictly monotonic for every metal. Across all systems and parametrizations, it remained below 0.15%. No nonstationary growth occurred within the analyzed *NPT* windows.

**Figure 2:**
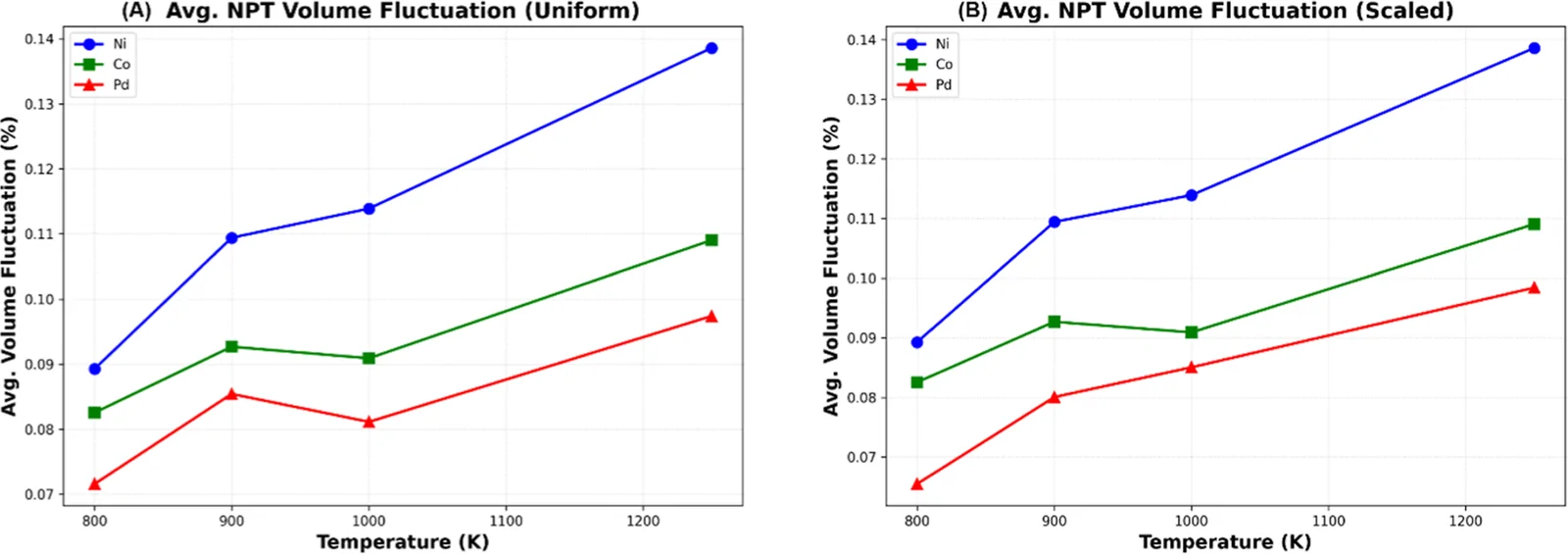
Temperature dependence of thermodynamic volume fluctuations. (A) Uniform and (B) sterically scaled hydrogen–metal parametrizations. Average normalized volume fluctuation amplitude σ_*V*_/⟨*V*⟩ is reported over the final 10 ps of *NPT* equilibration for Ni, Co, and Pd. Fluctuations remain below 0.15% under all tested conditions.

Figure 3A,B report the maximum volumetric swelling relative to hydrogen-free baselines. Swelling stayed below 0.09% and remained bounded for all metals and temperatures. The ordering Ni > Co > Pd was retained across the tested temperatures. No divergent replica behavior was observed.

**Figure 3:**
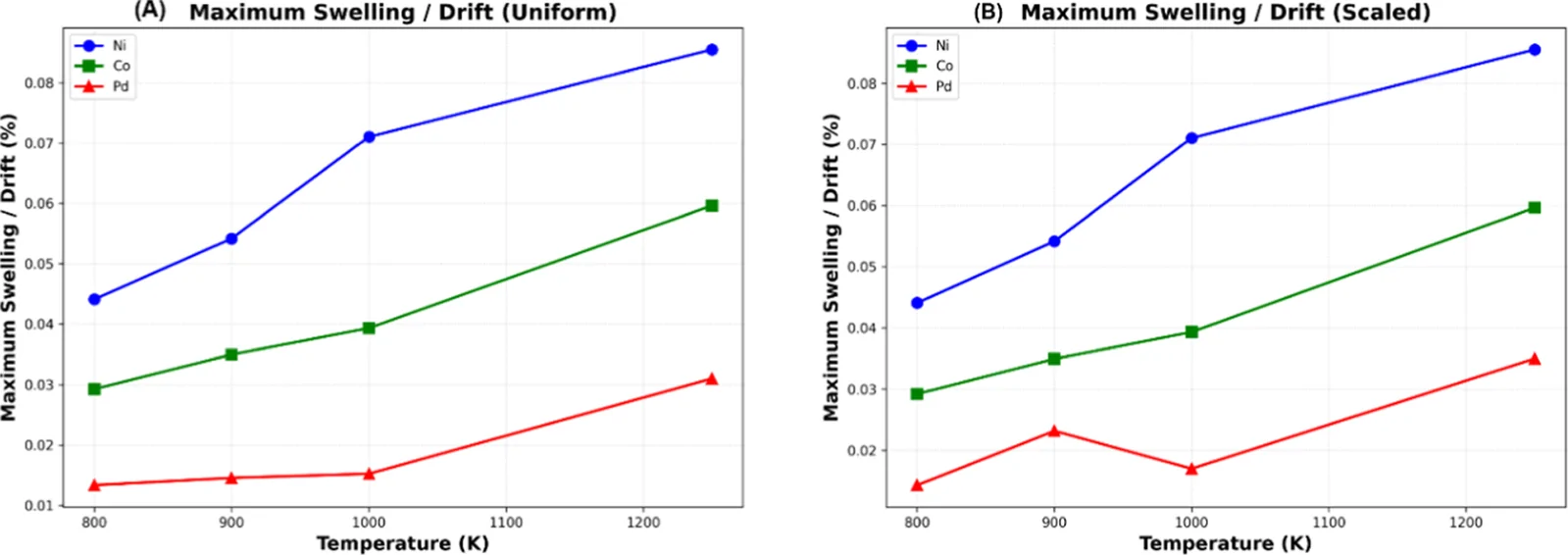
Maximum volumetric swelling induced by hydrogen insertion. Maximum relative volume change (*V*_H_ – *V*_0_)/*V*_0_ observed during *NPT* equilibration for Ni, Co, and Pd. Swelling remains bounded for all metals and temperatures.

### 3.2 Diffusive Linearity

Hydrogen transport was quantified using mean-squared displacement (MSD) analysis under *NVT* conditions following equilibration. Only trajectories satisfying the structural stability criteria in Section 3.1 were retained for diffusion analysis. Hydrogen MSDs showed linear time dependence over extended late-time windows for all metals and temperatures. Representative MSD trajectories at 1250 K are shown in Figure 4. No persistent departure from linear late-time diffusion was detected within the accepted fitting windows.

**Figure 4:**
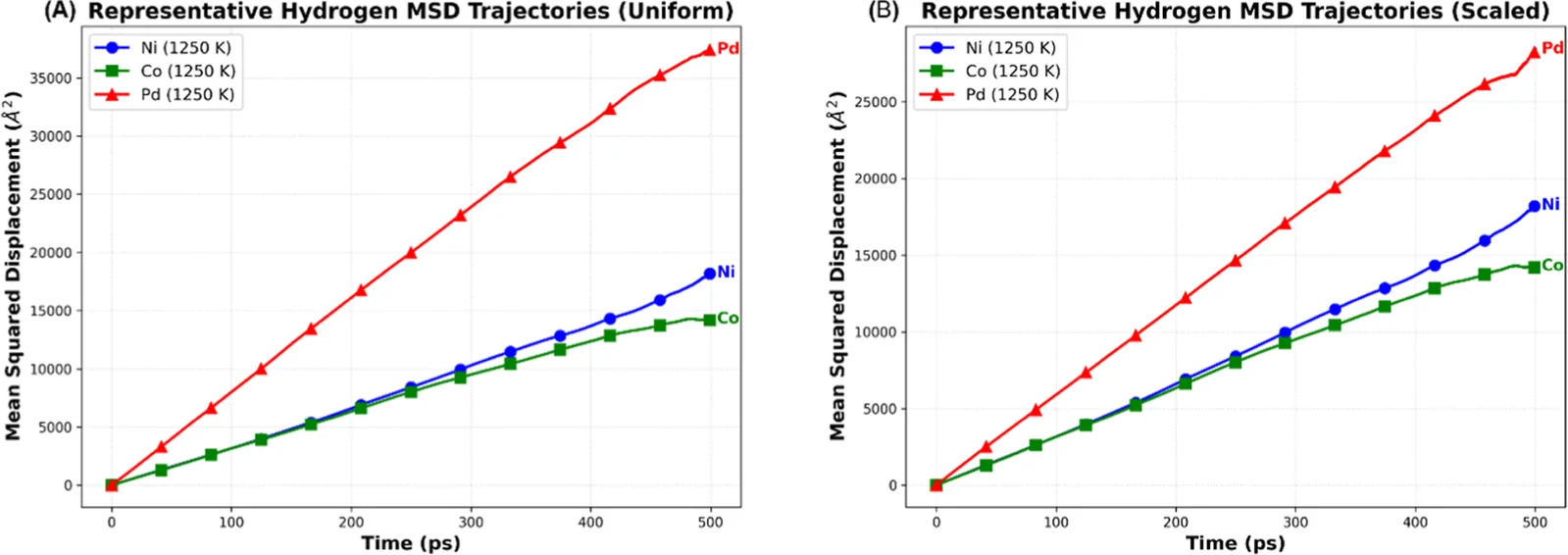
Representative hydrogen mean-squared displacement trajectories. Multiorigin MSD as a function of time at 1250 K for Ni, Co, and Pd under *NVT* conditions. Linear late-time behavior is observed for all systems.

Diffusion coefficients were extracted using adaptive late-time window fitting. Accepted fits satisfied *R* ^2^ ≥ 0.99, minimum window durations of 50 ps, and negligible slope drift under window refinement. Across all metal-temperature conditions and parametrizations, accepted fits showed strong linearity. The lowest reported minimum MSD *R* ^2^ was 0.999839 for uniform Pd at 900 K (Table 2). All reported group minima therefore exceeded 0.9998. Figure 5 summarizes the distribution of MSD linearity coefficients across all simulations.

**Table 2:** Hydrogen Diffusion Statistics from Three Independent Replicas^a^

Metal	Temp (K)	Model	*D*_mean_ (m^2^/s)	*D*_std_ (m^2^/s)	CV (%)	min *R* ^2^
Ni	800	Uniform/Scaled	1.810 × 10^–8^	2.230 × 10^–9^	12.32	0.999935
Ni	900	Uniform/Scaled	2.728 × 10^–8^	4.354 × 10^–9^	15.96	0.999922
Ni	1000	Uniform/Scaled	3.107 × 10^–8^	1.436 × 10^–9^	4.62	0.999932
Ni	1250	Uniform/Scaled	5.485 × 10^–8^	5.691 × 10^–9^	10.37	0.999909
Co	800	Uniform/Scaled	2.220 × 10^–8^	1.664 × 10^–9^	7.50	0.999948
Co	900	Uniform/Scaled	2.623 × 10^–8^	1.527 × 10^–9^	5.82	0.999935
Co	1000	Uniform/Scaled	3.440 × 10^–8^	2.927 × 10^–9^	8.51	0.999875
Co	1250	Uniform/Scaled	5.216 × 10^–8^	2.234 × 10^–9^	4.28	0.999872
Pd	800	Uniform	7.933 × 10^–8^	3.074 × 10^–9^	3.87	0.999927
Pd	800	Scaled	6.144 × 10^–8^	1.391 × 10^–9^	2.26	0.999947
Pd	900	Uniform	9.551 × 10^–8^	1.553 × 10^–8^	16.26	0.999839
Pd	900	Scaled	6.341 × 10^–8^	8.434 × 10^–9^	13.30	0.999925
Pd	1000	Uniform	1.001 × 10^–7^	9.658 × 10^–9^	9.64	0.999934
Pd	1000	Scaled	7.777 × 10^–8^	2.234 × 10^–9^	2.87	0.999902
Pd	1250	Uniform	1.331 × 10^–7^	1.544 × 10^–8^	11.60	0.999893
Pd	1250	Scaled	1.034 × 10^–7^	7.465 × 10^–9^	7.22	0.999843

a Mean self-diffusivity, standard deviation, coefficient of variation and minimum MSD linearity coefficient are reported for each metal-temperature condition. Ni and Co are listed as uniform/scaled because their hydrogen–metal parameters are identical in both parameterizations. Pd is reported separately for uniform and scaled models because σ_Pd_ differs between the two cases.

**Figure 5:**
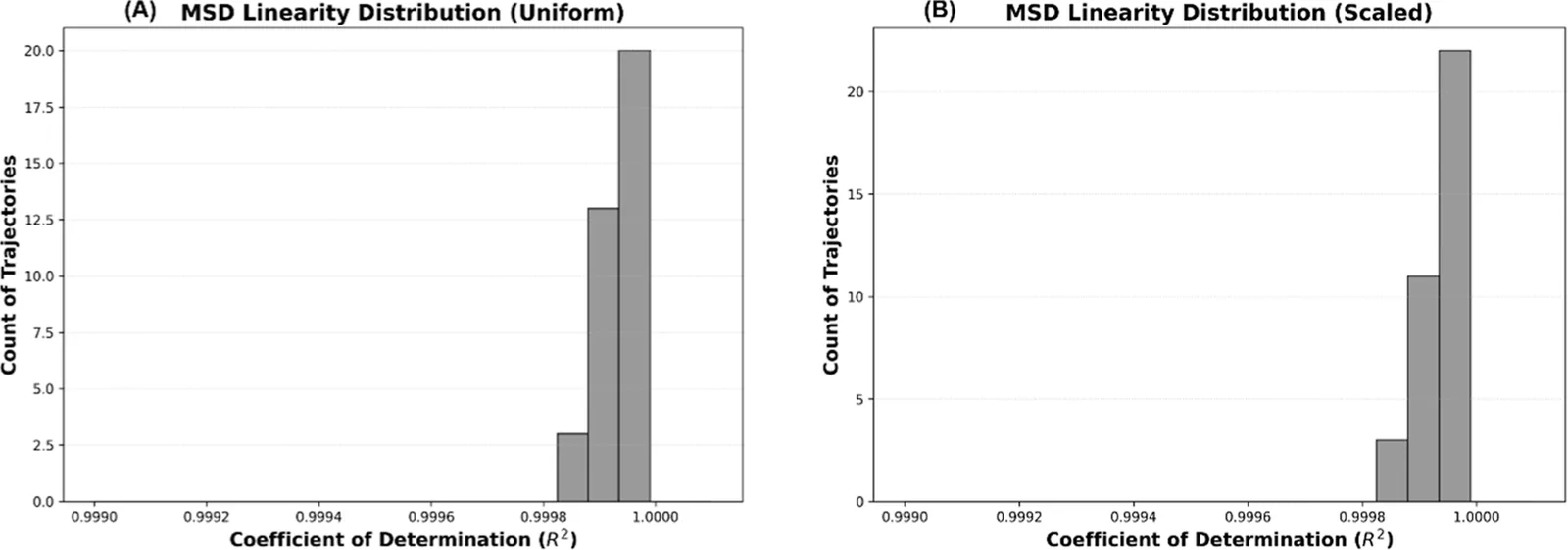
Distribution of MSD linearity coefficients. Histogram of coefficients of determination (*R* ^2^) obtained from linear fits to the late-time MSD regime across all simulations.

Statistical reproducibility was assessed using three independent hydrogen insertion replicas per metal-temperature condition. The maximum coefficient of variation was 16.26% (Table 2), demonstrating bounded but nonzero replica variability across insertion seeds.

### 3.3 Temperature-Dependent Diffusion

Temperature-dependent hydrogen self-diffusivities for Ni, Co, and Pd are shown in Figure 6A,B for the uniform and scaled parametrizations, respectively. Diffusion coefficients increased monotonically with temperature for all metals.

**Figure 6:**
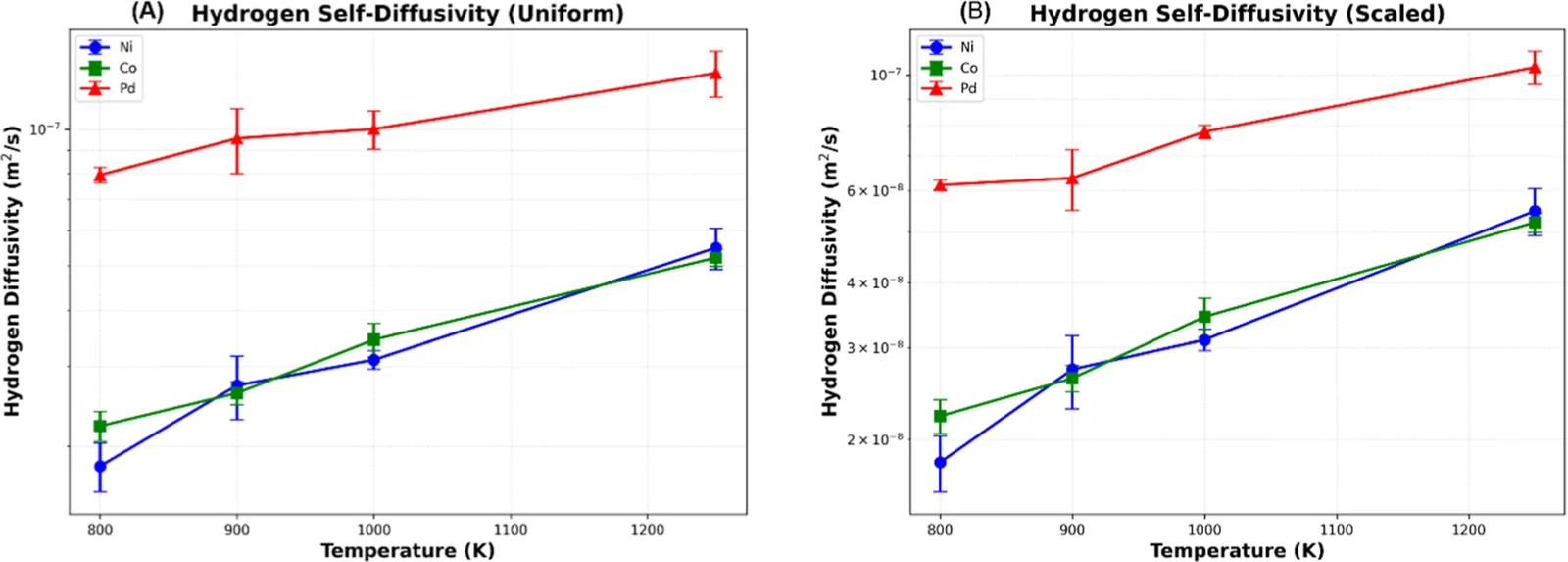
Temperature-dependent hydrogen self-diffusivity. Hydrogen diffusion coefficients for Ni, Co, and Pd from 800 to 1250 K for (A) uniform and (B) steric-scaled parametrizations. Data points represent the mean of three independent replicas; error bars indicate one standard deviation.

Across the full temperature range (800–1250 K), Pd consistently exhibited higher hydrogen mobility than Ni and Co. Ni and Co remained comparable and alternated slightly in ordering across temperature. The resulting comparative hierarchy is therefore *D*_Pd_ > *D*_Co_ ≈ *D*_Ni_, rather than a persistent Ni–Co ordering. This hierarchy was retained under both parametrizations.

### 3.4 Arrhenius Analysis

Arrhenius plots of diffusivity against inverse temperature are shown in Figure 7. Ni and Co give Arrhenius *R* ^2^ values of 0.9845 and 0.9857, respectively. Uniform Pd gives *R* ^2^ = 0.9761, while scaled Pd gives the weaker fit of *R* ^2^ = 0.9376 (Table 3). Regression robustness was examined using leave-one-temperature-out fitting in addition to the global regression described in Section 2.5. The resulting *Q* ranges were 0.1890–0.2112 eV for Ni, 0.1491–0.1891 eV for Co, 0.0817–0.0984 eV for uniform Pd, and 0.0787–0.1340 eV for scaled Pd. No prespecified regression-instability flag was triggered for Ni, Co, or uniform Pd. Scaled Pd remained the least constrained fit because its global *R* ^2^ is below 0.95 and its leave-one-temperature-out range is wider.

**Figure 7:**
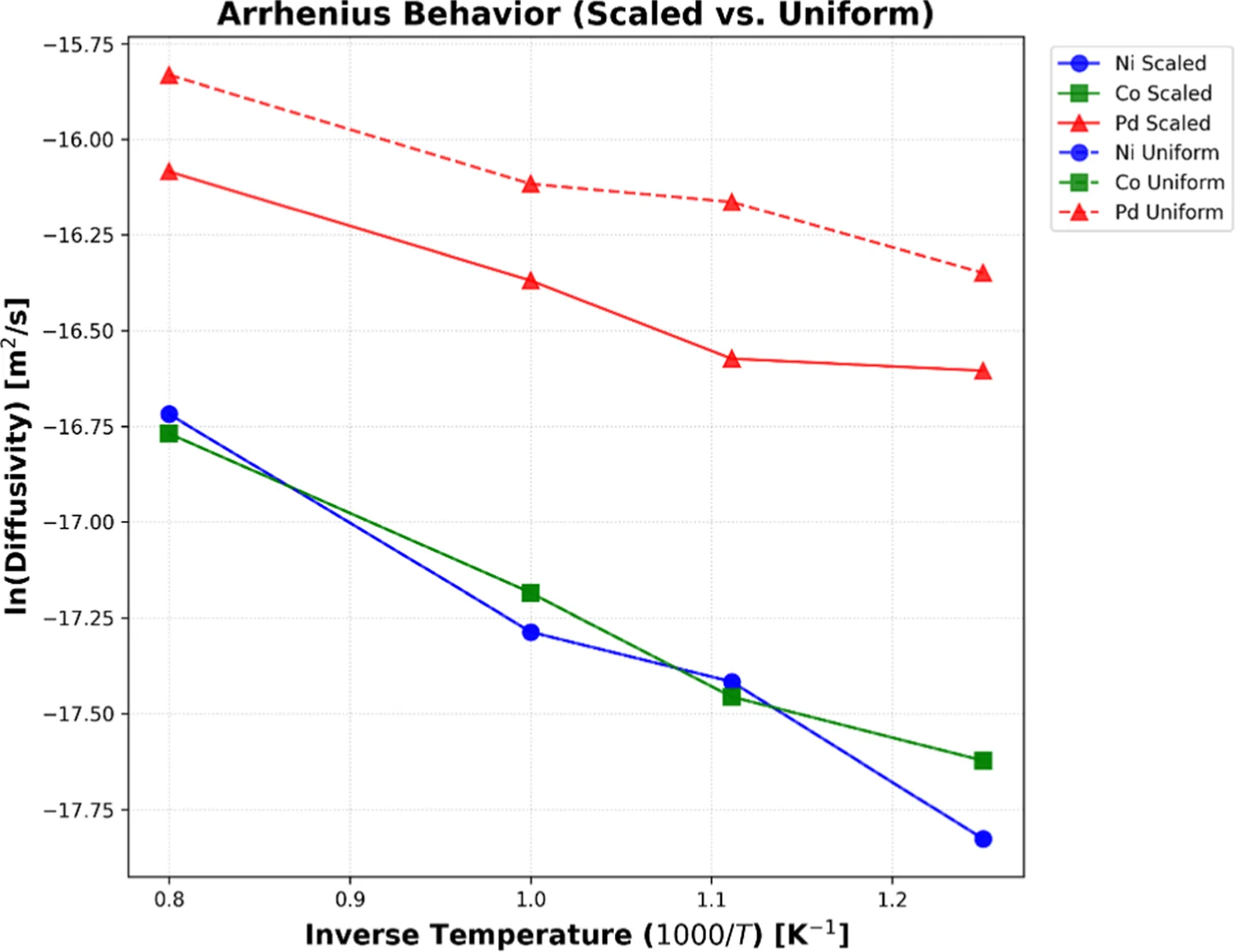
Arrhenius representation of hydrogen diffusion. Natural logarithm of diffusivity (ln *D*) plotted against inverse temperature (1000/*T*) for uniform (dashed) and steric-scaled (solid) parametrizations. Linear fits are shown for all metals.

**Table 3:** Extracted Arrhenius Transport Parameters^a^

Model	Metal	*Q* (eV)	95% CI for *Q* (eV)	*D*_0_ (m^2^/s)	*R* ^2^
Scaled	Ni	0.2060	0.127–0.285	3.6439 × 10^–7^	0.9845
Scaled	Co	0.1679	0.106–0.229	2.4259 × 10^–7^	0.9857
Scaled	Pd	0.1053	0.023–0.188	2.6671 × 10^–7^	0.9376
Uniform	Ni	0.2060	0.127–0.285	3.6439 × 10^–7^	0.9845
Uniform	Co	0.1679	0.106–0.229	2.4259 × 10^–7^	0.9857
Uniform	Pd	0.0960	0.050–0.142	3.1931 × 10^–7^	0.9761

a Activation energies *Q*, 95% confidence intervals, pre-exponential factors *D*_0_ and coefficients of determination *R* ^2^ were obtained from linear regression of ln­(*D*) against 1000/*T*.

Diffusivity increases monotonically with temperature for every system (Figure 6). Accepted late-time MSD fits also remain linear under the criteria defined in Section 3.2, with the corresponding minimum MSD *R* ^2^ values reported in Table 2. MSD linearity establishes diffusive late-time behavior but does not independently establish Arrhenius linearity.

Activation energies extracted from linear regression are summarized in Table 3. The fitted Pd shift from 0.0960 to 0.1053 eV is not treated as statistically resolved because the regression confidence intervals overlap substantially. The primary finite-temperature scaling evidence is therefore the systematic reduction in Pd diffusivity at every tested temperature (Figure 6). Ni and Co remain identical across both parametrizations because their hydrogen–metal interaction parameters are unchanged (Table 3).

### 3.5 Effect of Steric Scaling

To isolate the effect of steric scaling, Arrhenius activation energies were extracted for the uniform and scaled parametrizations using identical procedures. Figure 8 compares them for the three metals. For Ni and Co, where the hydrogen–metal interaction parameters are unchanged, the extracted activation energies remain identical (Table 3). For Pd, increasing σ_Pd_ from 1.45 to 1.50 Å shifts the fitted activation energy from 0.0960 to 0.1053 eV (Table 3; Figure 8). The 95% confidence intervals overlap, so the Arrhenius analysis does not resolve this fitted separation statistically. The stronger finite-temperature evidence is the systematic decrease in Pd diffusivity at every tested temperature (Figure 6). The scaled-Pd Arrhenius fit remains the least constrained of the four fitted parameter sets, as discussed in Section 3.4 and shown in Figure 7.

**Figure 8:**
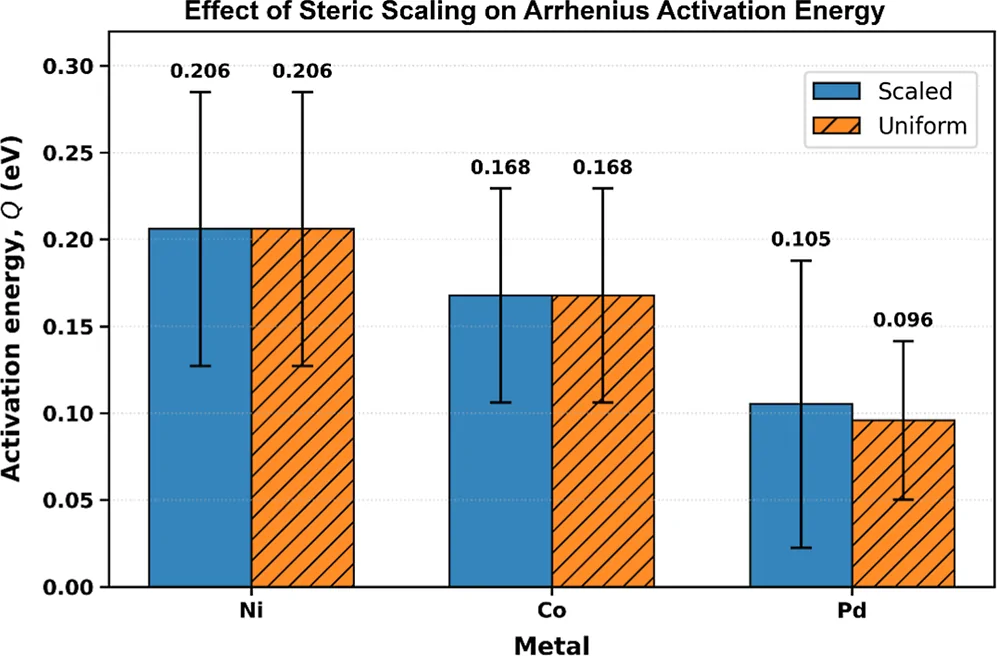
Effect of steric scaling on fitted Arrhenius activation energy. Bars show activation energies obtained from the four-temperature regressions in Table 3. Error bars show the corresponding 95% regression confidence intervals. The overlapping Pd intervals indicate that the fitted uniform-to-scaled *Q* shift is not statistically resolved.

The geometric basis for scaling only Pd is given by the confinement ratios in Table S1. Increasing σ_Pd_ raises the confinement ratio from 0.84 to 0.87, while Ni and Co retain the baseline value near 0.92.

A 5.0 at. % Pd sensitivity test retained lower scaled-Pd diffusivity at all four temperatures, with *D*_scaled_/*D*_uniform_ = 0.680–0.755. Maximum swelling remained below 0.12%. Arrhenius fits gave *Q* = 0.1143 eV for uniform Pd and 0.1161 eV for scaled Pd. These results test persistence of the scaling direction rather than concentration-independent kinetics. Full values are reported in Section S.8, Tables S3 and S4, and Figure S1.

### 3.6 Pathway-Resolved Static Migration Barriers

High-frequency trajectory analysis first tested whether the nearest-neighbor O-site end point family remained dynamically relevant for Ni and Co. At 2 fs sampling, 97.5–99.0% of detected fine-cadence O-site changes had nearest-neighbor end points. Decimating the same trajectories to 0.1 ps reduced this fraction to 48.5–61.3%. The reduction demonstrates temporal aliasing when elementary hops are classified from the 0.1 ps production cadence. It does not invalidate that cadence for long-time MSD extraction. Frequent passage through the associated tetrahedral region was also resolved. These observations support the nearest-neighbor O–O end point family without identifying T as the transition state.

Local-stability calculations then resolved the static interstitial topology. Ideal O and T positions form separate local minima in Ni and Co. T therefore cannot represent the Ni or Co saddle under the present interaction model. For both Pd parametrizations, the ideal O position is locally unstable. Stable off-center O* minima instead form along the crystallographic O–T corridors.

Pathway-resolved CI-NEB gives static barriers of 0.1516 eV for Ni, 0.1294 eV for Co, 0.0234 eV for uniform Pd, and 0.0398 eV for scaled Pd. Multiple initial bands and image-count refinement gave stable barrier values. Full local-stability, pathway, and force-convergence diagnostics are provided in Section S.7. The resulting static ordering Ni > Co > Pd_scaled_ > Pd_uniform_ matches the Arrhenius ordering in Table 3. Figure 9 shows the final 0 K energy landscapes, while Table S2 compares the static and finite-temperature barriers.

**Figure 9:**
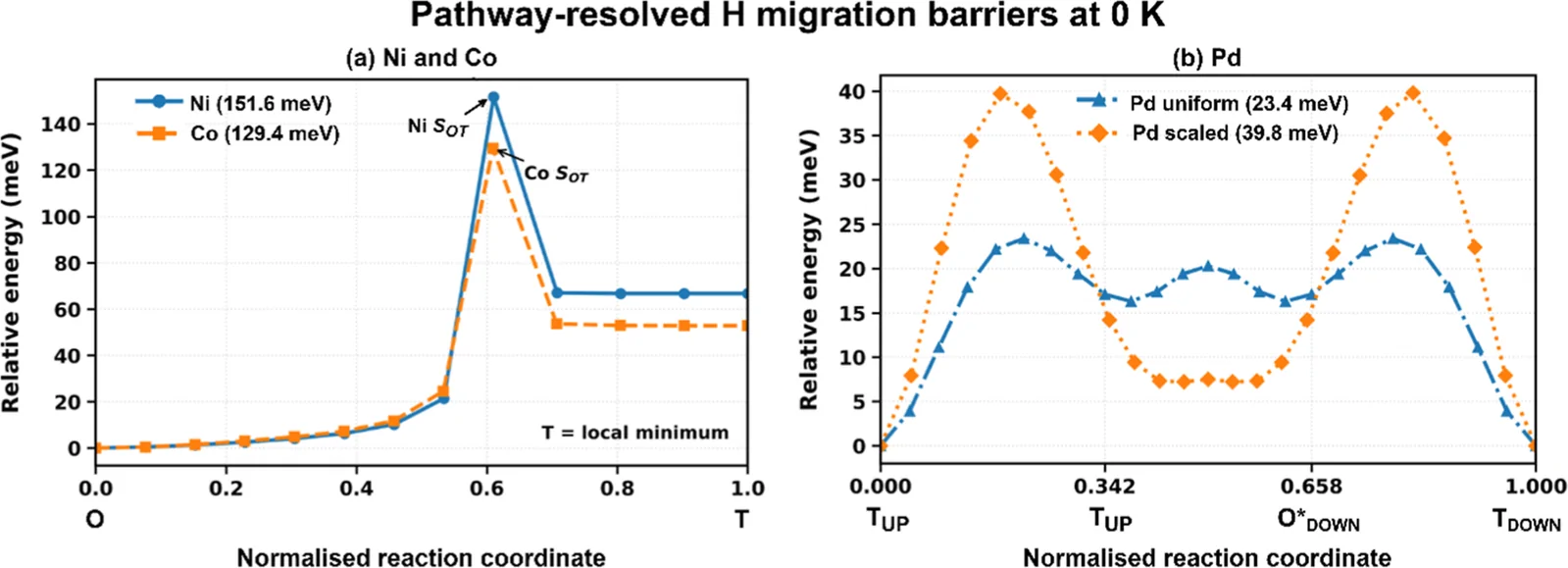
Pathway-resolved single-H CI-NEB migration barriers at 0 K. (a) Ni and Co migration between verified O and T local minima, with *S*_OT_ identifying the intervening saddle. T is a local minimum rather than the transition state. (b) Uniform and scaled Pd migration along the verified T_UP_ → O_UP_^*^ → O_DOWN_^*^ → T_DOWN_ network. The static barriers are 0.1516, 0.1294, 0.0234, and 0.0398 eV for Ni, Co, uniform Pd and scaled Pd, respectively. Energies are referenced to the initial stable basin. Panels use independent vertical scales to resolve the lower Pd barriers.

## 4 Discussion

### 4.1 Structural Integrity under Baseline Hydrogen Loading

The central hypothesis of this study is that hydrogen transport can be modulated through the H–metal steric parameter σ while retaining the host EAM description and LJ well depth. The uniform and scaled responses in Sections 3.3–3.6 test whether this modification changes Pd transport without destabilizing the FCC host lattice.

Across all metals and temperatures investigated (800–1250 K), the FCC lattices remain structurally stable at the baseline finite concentration of approximately 1.1 at. % H/metal. Normalized volume fluctuations remain below 0.15% (Figure 2), and maximum volumetric swelling remains below 0.09% relative to hydrogen-free baselines (Figure 3). No nonstationary drift, lattice collapse, or runaway expansion is observed in any trajectory. These bounds apply to the monitored volume response and do not constitute a measurement of elastic constants or mechanical strength.

The swelling hierarchy Ni > Co > Pd is retained across the tested temperatures (Figure 3). This ordering is specific to the present parametrization and is not treated as a universal FCC swelling relation. Increasing σ_Pd_ does not produce an unbounded or systematic structural instability. The steric intervention therefore changes the migration landscape while the monitored lattice-volume response remains bounded.

### 4.2 Steric Scaling as a Controlled Modifier of Migration Barriers

In the uniform model, Pd shows a fitted activation energy of *Q*_Pd_ = 0.096 eV (Table 3). Increasing σ_Pd_ from 1.45 Å to 1.50 Å gives *Q*_Pd_ = 0.1053 eV (Table 3; Figure 8). This shift falls within the regression confidence interval, so the Arrhenius fit does not resolve the barrier change. The robust finite-temperature response is the systematic reduction in Pd diffusivity at every tested temperature (Figure 6). The scaled-Pd four-temperature fit remains less constrained than the other fits, as quantified in Section 3.4 and Table 3. The static pathway analysis provides an independent test of the scaling direction. Increasing σ_Pd_ raises the verified single-H 0 K barrier from 0.0234 to 0.0398 eV (Figure 9; Table S2). The finite-temperature diffusivity response and the static pathway calculation therefore agree on the direction of the Pd steric-scaling effect without requiring equal barrier magnitudes.

For Ni and Co, the hydrogen–metal interaction parameters are unchanged between the two parametrizations. Ni and Co therefore serve as unchanged controls rather than independent tests of scaling. Their identical parametrizations explain the absence of a model-dependent shift in *Q* or *D*_0_ (Table 3). At 5.0 at. % H/metal, scaled Pd also remains less diffusive than uniform Pd at every tested temperature (Figure S1; Tables S3 and S4). This result establishes persistence of the scaling direction at the higher tested loading but does not establish concentration-independent kinetics. Within the present pairwise model, σ_Pd_ sets the characteristic H–Pd separation in the short-range interaction. Its transport effect is established by the present sensitivity calculations rather than inferred as a universal hydrogen radius. The evidence is the persistent diffusivity reduction and the independently resolved static-barrier increase.

### 4.3 Geometric Contribution to the FCC Transport Hierarchy

For the baseline uniform parametrization, the fitted Arrhenius barriers follow *Q*_Ni_ > *Q*_Co_ > *Q*_Pd_ with *Q*_Ni_ = 0.2060 eV, *Q*_Co_ = 0.1679 eV, and *Q*_Pd_ = 0.0960 eV (Table 3). This ordering is consistent with the observed *D*_Pd_ > *D*_Co_ ≈ *D*_Ni_ hierarchy over 800–1250 K (Figure 6). Diffusivity also depends on *D*_0_, so the transport ordering is not attributed to *Q* alone. The geometric confinement metric provides one descriptor of this trend. Under the uniform parametrization, α ≈ 0.92 for Ni and Co, then 0.84 for Pd (Table S1). The lower Pd value identifies weaker geometric confinement relative to the ideal FCC reference geometry. It does not establish the stable interstitial topology.

This distinction is required because the local-stability calculations show system-dependent pathways. Ideal O and T are separate local minima for Ni and Co, whereas ideal O is unstable for Pd (Section 3.6). The confinement ratio is therefore a geometric screening metric, while the actual migration topology and saddle energies are determined by the local-stability and CI-NEB calculations (Figure 9).

Within this hybrid EAM/LJ framework, geometric confinement is a resolved control variable rather than the sole determinant of migration energetics. Independent Ni–Mn calculations showed that lattice expansion lowered hydrogen migration barriers, while repulsive Mn–H interactions increased them.^27^ DFT calculations across transition metals also linked hydrogen solution and diffusion energetics to changes in electronic d-band descriptors.^67^ Finite-temperature Pd simulations reported an activation energy of 0.16 eV with mobile host atoms and 0.20 eV with fixed Pd.^68^ For FCC γ-Fe, calculations showed that changing magnetic ordering altered the calculated hydrogen migration barriers.^69^ Together, these studies show that hydrogen migration energetics can depend on geometry, H–metal interactions, electronic structure, host dynamics, and magnetic state. The present H–metal LJ description does not resolve electronic or magnetic contributions explicitly.

The present Arrhenius barriers are not treated as quantitative reproductions of reference transport kinetics. Pure-Ni simulations by Ito et al. reported *Q* = 0.42 eV, close to an experimental value of 0.44 eV.^27^ Both values exceed the present *Q*_Ni_ = 0.2060 eV. Tang et al.^68^ obtained 0.16 eV for hydrogen diffusion with mobile Pd atoms. This value also exceeds the present Pd estimates of 0.0960 and 0.1053 eV. No causal mechanism is assigned to these offsets. Validation is therefore based on transport ordering, temperature dependence, MSD linearity, regression quality, and bounded lattice response rather than absolute barrier agreement.

Steric scaling therefore isolates one model-level geometric contribution and should not be interpreted as a universal crystallographic law. The corrected static barriers follow Ni > Co > Pd_scaled_ > Pd_uniform_ (Figure 9; Table S2), matching the Arrhenius ordering in Table 3. Static CI-NEB and Arrhenius *Q* represent distinct quantities, so numerical equality is not imposed. A previous FCC-Ni MD study similarly reported different activation energies from MSD-based Arrhenius analysis and NEB calculations, providing an independent precedent for treating these quantities separately.^70^ The former Ni and Co static-over-dynamic excess was removed by correcting the static pathway, while Section 3.4 did not identify Arrhenius regression instability sufficient to explain it. A dominant finite-concentration effect is therefore not required for that discrepancy, although concentration effects on absolute kinetics remain unresolved.

Pd alone was scaled on confinement-ratio grounds. Pd has the largest lattice parameter and the weakest steric confinement, with α = 0.84 compared with 0.92 for Ni and Co (Table S1). Raising σ_Pd_ from 1.45 to 1.50 Å increases α to 0.87 and partially restores confinement relative to the smaller-lattice metals. Ni and Co were therefore retained at the baseline σ = 1.45 Å and used as unchanged controls.

### 4.4 Implications for Mechanically Coupled Molecular Dynamics

A primary motivation of this work is the development of hydrogen transport models suitable for large-scale, mechanically coupled MD simulations. The present validation addresses three prerequisites for using the transport model in later coupled calculations.

First, structural admissibility is maintained. Hydrogen insertion produces bounded swelling and stationary lattice-volume response under the tested conditions (Figures 2 and 3). Second, computational scalability is maintained through the hybrid EAM/LJ formulation, consistent with the larger atom counts and longer time scales accessible to classical LJ models relative to first-principles treatments.^22^ Third, accepted trajectories retain linear late-time MSD behavior (Figures 4 and 5), with the corresponding reproducibility statistics reported in Table 2. Diffusivity increases monotonically with temperature for every system (Figure 6). Ni, Co and uniform Pd retain stable Arrhenius fits, while scaled Pd remains less tightly constrained (Figure 7; Table 3). The pathway-resolved static calculations provide a separate 0 K validation of migration topology and scaling direction (Figure 9; Table S2).

Coupled hydrogen-fracture formulations explicitly combine deformation, hydrogen transport, trapping, and fracture, making the transport description part of the predicted crack response.^71^ Together, these checks establish a bounded bulk transport framework for subsequent hydrogen-stress coupling calculations. They do not constitute direct validation of stress-driven redistribution, trapping, plasticity, or fracture, which are outside the present simulations.

### 4.5 Limitations and Scope of Validity

The framework isolates intrinsic bulk hydrogen diffusion in defect-free FCC lattices at a baseline finite concentration of approximately 1.1 at. % H/metal. Surface chemistry, hydride formation, hydrogen clustering, dislocation interactions, and quantum nuclear effects are not represented. Quantum effects contribute to hydrogen isotope diffusion in metals, and density functional theory (DFT) derived barriers depend on the electronic-structure treatment.^72–76^ The extracted activation energies should therefore be interpreted as effective barriers governing thermally activated interstitial migration within the validated temperature and concentration range. The static CI-NEB calculations represent single-H 0 K potential-energy landscapes. They do not include finite-temperature free-energy contributions or quantum nuclear corrections. Static and Arrhenius barriers are therefore compared by pathway ordering and scaling direction rather than by assuming numerical equality.

The stability bounds reported here apply to bulk, defect-free, fully periodic lattices. They should not be transferred directly to free surfaces or nanoparticles. Pd nanoparticle calculations show size- and shape-dependent hydrogen response associated with free-surface strain and surface stress.^43^ Surface and finite-size relaxation can therefore alter hydrogen-induced lattice response outside the present periodic-bulk domain. The higher-loading test in Sections 3.5 and S.8 is a Pd-only sensitivity check of the σ-scaling direction, not a full concentration-dependent transport model for all three metals.

The 1.1 at. % production state is therefore not treated as an infinite-dilution limit. Concentration effects on absolute Arrhenius barriers remain unquantified for Ni and Co. The 5.0 at. % Pd calculations test whether the σ-scaling direction persists at a higher loading. They do not establish concentration-independent transport kinetics. Correcting the static pathways removes the former excess of static over Arrhenius barriers for Ni and Co. A dominant finite-concentration effect is therefore not required to explain that specific discrepancy. This conclusion does not exclude concentration-dependent transport outside the tested conditions.

The steric-scaling workflow can be extended in principle to other FCC metals, including Cu, Ag, and Au. Such an extension requires more than substituting the metal species. The metallic potential, H–metal cross-interaction, local interstitial topology, Arrhenius response, and lattice-stability bounds must each be revalidated. The present study therefore demonstrates the approach for Ni, Co, and Pd, while treating extension to other FCC metals as feasible but not established here. By explicitly defining this scope, the present study bounds the interaction framework to a specific domain of validity, aligning with the statistically averaged MD approaches recommended for hydrogen transport.^8^

## 5 Conclusion

A sterically informed hybrid EAM/Lennard-Jones framework was evaluated for hydrogen transport in FCC Ni, Co, and Pd over 800–1250 K. At the baseline 1.1 at. % H/metal state volume fluctuations remained below 0.15% and swelling below 0.09%. Accepted trajectories retained linear late-time MSD behavior and diffusivity followed *D*_Pd_ > *D*_Co_ ≈ *D*_Ni_. Arrhenius fitting gave 0.2060 eV for Ni, 0.1679 eV for Co, 0.0960 eV for uniform Pd, and 0.1053 eV for scaled Pd and the Pd *Q* shift is not statistically resolved. Scaling nevertheless reduced Pd diffusivity at every tested temperature and retained this direction at 5.0 at. % H/metal. Pathway-resolved single-H calculations identified *T* as a local minimum in Ni and Co and ideal O as unstable in Pd and the final static barriers are 0.1516, 0.1294, 0.0234, and 0.0398 eV for Ni, Co, uniform Pd, and scaled Pd, respectively.

Correcting the static pathways removes the former Ni and Co static-overdynamic discrepancy without requiring Arrhenius failure or a dominant concentration effect. Concentration effects on absolute kinetics remain unresolved. The framework therefore provides a bounded bulk transport basis for subsequent mechanically coupled MD while stress-driven redistribution, trapping, plasticity and fracture require separate validation.

## Supplementary Material



## Notes

Hydrogen–metal steric confinement parameters and geometric screening metrics; hydrogen insertion protocol and replica initialization; diffusion trajectory sampling and multiorigin MSD analysis; diffusion-coefficient extraction and Arrhenius regression robustness; statistical reproducibility and structural stability diagnostics; LAMMPS execution and postprocessing pipeline; high-frequency Ni and Co migration-pathway audit; local stability of ideal octahedral and tetrahedral interstitial sites; independent verification of Pd off-center O* minima; segmented Ni and Co CI-NEB calculations; Pd verified-network CI-NEB calculations; image-count and force-convergence analyses; higher hydrogen-loading sensitivity of Pd transport and lattice swelling; supporting equations, tables and figure (PDF)
