# Supplementary material for: Steric Scaling of Hydrogen Transport in FCC Transition Metals for Mechanically Consistent Molecular Dynamics Simulations
Source: ACS Omega. 2026 Oct 15;11(42):64724–36. doi: 10.1021/acsomega.6c05027 (PMC13717784; doi:10.1021/acsomega.6c05027)
Supplement: Supplementary file 1 [file ao6c05027_si_001.pdf]

Supporting Information

Steric Scaling of Hydrogen Transport in FCC Transition Metals for Mechanically Consistent Molecular Dynamics Simulations

Emmanuel Peters-Teke Tebo [11], Sina Karimzadeh [12], Nyuytifo Emmanuel Wiykiynyuy [13], Tien-Chien Jen[*14]

[1] *Department of Mechanical Engineering Science, University of Johannesburg, Gauteng, 2006, South Africa*

*Corresponding author email: tjen@uj.ac.za

Contents

S.1 Hydrogen–Metal Steric Confinement Parameters 2
S.2 Hydrogen Insertion and Replica Initialization 2
S.3 Diffusion Trajectory Sampling 2
S.4 Diffusion Coefficient Extraction and Temperature Sampling 2
S.5 Statistical Reproducibility Criteria and Structural Stability Diagnostics 3
S.6 LAMMPS Execution and Post-Processing Pipeline 3
S.7 Pathway Topology, Local Stability, and Static Migration-Barrier Validation 3
S.7.1 High-Frequency Ni/Co Pathway Audit 3
S.7.2 Local Stability of Ideal O and T Reference Positions 3
S.7.3 Independent Verification of Pd O* Minima 4
S.7.4 Ni and Co Segmented CI-NEB 4
S.7.5 Pd Verified-Network CI-NEB 4
S.7.6 Final Image and Force Convergence 4
S.8 Concentration Sensitivity at Higher H Loading 5

[1] Email: 221191307@student.uj.ac.za
[2] Email: skarimzadeh@uj.ac.za
[3] Email: nyuytifo@gmail.com
[*4] Corresponding Author, Email: tjen@uj.ac.za

S.1 Hydrogen–Metal Steric Confinement Parameters

Steric scaling of hydrogen–metal interactions was implemented through an adjustment of the Lennard–Jones size parameter σ for palladium. The Lennard–Jones minimum interaction distance was calculated from Eq. S1,

$$r_{\min} = 2^{1/6}\sigma, \quad \text{(S1)}$$

where σ is the Lennard–Jones size parameter. The confinement ratio, α, was then calculated using Eq. S2 by comparing $r_{\min}$ with the octahedral nearest-neighbour distance, $a/2$, of the host FCC lattice,

$$\alpha = \frac{r_{\min}}{a/2} \quad \text{(S2)}$$

Steric confinement ratios were not enforced to be identical across metals. The Pd size parameter was increased to partially compensate for its larger FCC lattice parameter, while Ni and Co retained the baseline parameterisation. The confinement ratio is used as a geometric screening metric and does not determine local-site stability or migration topology. **Table S1** reports the resulting a, σ, $r_{\min}$ $a/2$ and α values.

Table S1. Hydrogen–Metal Steric Confinement Metrics

Metal	Parameter Set	a (Å)	σ (Å)	$r_{\min}$ (Å)	$a/2$ (Å)	α
Ni	Uniform / Scaled	3.52	1.45	1.63	1.76	0.92
Co	Uniform / Scaled	3.55	1.45	1.63	1.78	0.92
Pd	Uniform	3.89	1.45	1.63	1.95	0.84
Pd	Scaled	3.89	1.50	1.68	1.95	0.87

S.2 Hydrogen Insertion and Replica Initialization

Hydrogen atoms were inserted randomly within a central cubic region excluding one lattice spacing from each periodic boundary. The baseline production systems contained 100 H atoms and 8788 metal atoms, corresponding to approximately 1.1 at.% H/metal. This state is treated as a low finite-concentration baseline rather than an infinite-dilution limit. For each metal–temperature condition, three statistically independent replicas were generated using fixed hydrogen insertion seeds: 15485863; 32452843; 49979687. The higher-loading Pd sensitivity calculations used 440 H atoms in the same metal supercell, corresponding to 5.0 at.% H/metal. Details are provided in Section S.8.

S.3 Diffusion Trajectory Sampling

Hydrogen trajectories were recorded during NVT production runs using atom-style dumps containing periodic image flags. Hydrogen coordinates and box dimensions were stored every 200 timesteps. With the 0.5 fs production timestep, the resulting trajectory interval was 0.1 ps.

Unwrapped coordinates reconstructed from periodic image counters were used for the long-time multi-origin MSD analysis. The 0.1 ps cadence is sufficient for the diffusion calculation but can alias multiple elementary hops when used to classify individual migration events. A separate high-frequency pathway audit therefore stored hydrogen coordinates every 2 fs for selected Ni and Co trajectories. Those calculations are described in Section S.7.

S.4 Diffusion Coefficient Extraction and Temperature Sampling

Hydrogen MSD was computed using a multi-origin approach and diffusion coefficients were extracted using automated late-time window scanning. Candidate windows were required to satisfy $R^2 \geq 0.99$ and a minimum duration of 50 ps and negligible slope variation under window refinement. Baseline

finite-concentration simulations were conducted at 800, 900, 1000, and 1250 K and the higher-loading Pd sensitivity test used the same four temperatures. Arrhenius robustness was assessed independently of MSD linearity, and the audit included the global four-temperature regression and bootstrap resampling and leave-one-temperature-out fitting and adjacent-temperature apparent slopes. The leave-one-temperature-out Q ranges were 0.1890–0.2112 eV for Ni, 0.1491–0.1891 eV for Co, 0.0817–0.0984 eV for uniform Pd, and 0.0787–0.1340 eV for scaled Pd.

No prespecified regression-instability flag was triggered for Ni or Co or uniform Pd and scaled Pd remained the least constrained fit because its global $R^2 = 0.9376$ and its leave-one-temperature-out barrier range was weaker than those of the other systems. MSD linearity and Arrhenius linearity were therefore treated as separate diagnostics. The regression audit supports the Ni, Co, and uniform-Pd four-temperature fits, while scaled Pd remains the least statistically constrained fit under the tested temperature set.

S.5 Statistical Reproducibility Criteria and Structural Stability Diagnostics

Statistical reproducibility was assessed across three independent insertion replicas for each metal–temperature condition. The maximum observed diffusivity coefficient of variation in the baseline dataset was 16.26%. Hydrogen-free simulations were performed under matching conditions to establish the lattice-volume reference. Volume statistics and centrosymmetry-parameter histograms were evaluated during equilibration.

Across the baseline H-loaded simulations, normalised volume fluctuations remained below 0.15%, while maximum volumetric swelling relative to the hydrogen-free reference remained below 0.09%. These quantities were used as structural-stability diagnostics rather than measurements of elastic constants or mechanical strength.

S.6 LAMMPS Execution and Post-Processing Pipeline

All simulations were executed using LAMMPS with the hybrid EAM/Lennard–Jones interaction model defined in the main manuscript. Unwrapped hydrogen trajectories were reconstructed using periodic image counters and instantaneous box dimensions. Diffusion, Arrhenius, structural-stability, pathway-topology, local-stability, and NEB analyses were performed using automated post-processing workflows. The same H–metal parameter sets were retained between the production MD calculations and the corresponding static pathway calculations.

S.7 Pathway Topology, Local Stability, and Static Migration-Barrier Validation

S.7.1 High-Frequency Ni/Co Pathway Audit

The relevant Ni and Co endpoint family was tested before recalculating static barriers. Dedicated 10 ps trajectories were generated at 1000 and 1250 K using three independent replicas per condition and the baseline 100-H inventory and hydrogen coordinates were stored every 2 fs. At fine sampling, 99.0% and 97.5% of confirmed Ni O-site changes had nearest-neighbour endpoints at 1000 and 1250 K and the corresponding Co fractions were 98.8% and 98.0%. Decimating the same trajectories to the 0.1 ps production cadence reduced the apparent nearest-neighbour fractions to 61.3% and 48.5% for Ni and 61.3% and 49.7% for Co.

Frequent passage through the associated tetrahedral region was also resolved and the audit therefore retained the nearest-neighbour O–O endpoint family for Ni and Co while demonstrating that the 0.1 ps cadence aliases elementary-hop topology. The audit does not invalidate that cadence for long-time MSD extraction.

S.7.2 Local Stability of Ideal O and T Reference Positions

Single-H 0 K stability calculations used a 6×6×6 fully periodic FCC cell containing 864 metal atoms and one H atom and the box was fixed while all atoms were allowed to relax. Local stability was tested using signed perturbations along 26 low-index directions around the ideal O and T reference positions.

For Ni and Co, both ideal O and T behaved as restoring local minima and the relaxed T–O energy differences were 0.066738 eV for Ni and 0.052867 eV for Co, so T cannot be the Ni or Co migration saddle under this interaction model. For uniform Pd, the ideal O position was locally unstable with negative directional curvature and an off-centre branch 8.081 meV below ideal O and for scaled Pd the ideal O position was also locally unstable with an off-centre branch 1.030 meV below ideal O.

The deepest sampled Pd off-centre branches were aligned with the ⟨111⟩ O–T corridors and these states are denoted O*. The minimum frozen-host directional curvatures at ideal O were −0.11674 eV $Å^{-2}$ for uniform Pd and −0.05204 eV $Å^{-2}$ for scaled Pd.

S.7.3 Independent Verification of Pd O* Minima

The corridor-directed Pd O* states were subsequently perturbed in all 26 signed low-index directions and fully relaxed and all tested perturbations returned to the corresponding O* basin. The minimum directional curvatures were +0.14041 eV $Å^{-2}$ for uniform Pd and +0.08738 eV $Å^{-2}$ for scaled Pd and the frozen-host H Hessian was positive definite for both parameterisations. The verified T position remained lower in energy than O* by approximately 0.01628 eV for uniform Pd and 0.00700 eV for scaled Pd and accordingly ideal O was not used as a Pd NEB endpoint in the corrected pathway calculations.

S.7.4 Ni and Co Segmented CI-NEB

The corrected Ni and Co static topology was treated as O→S_{OT}→T→$S_{TO'}$→O′. CI-NEB calculations were therefore performed separately for the O-to-T and symmetry-related T-to-O segments. Three independent initial bands were tested for each segment, giving twelve pathway calculations across Ni and Co.Each calculation used thirteen replicas and an NEB force tolerance of 0.01 eV $Å^{-1}$ during pathway identification and all three initialisations converged to the same saddle families. The pathway audit yielded O-referenced barriers near 0.1515 eV for Ni and 0.1294 eV for Co and the forward–reverse energy differences independently reproduced the relaxed T–O differences from Section S7.2. These results demonstrate that the former large Ni and Co barriers arose from the previous direct high-symmetry path construction rather than from the relevant O-site endpoint family.

S.7.5 Pd Verified-Network CI-NEB

Pd was treated using the verified T_{UP}→O^*_{UP}→O^*_{DOWN}→T_{DOWN} network and segmented calculations tested T_{UP}→O^*_{UP}, O^*_{UP}→O^*_{DOWN}, and O^*_{DOWN}→T_{DOWN}. Three independently initialised bands were used for every segment and whole-route T-to-T controls were also calculated. A total of twenty-six Pd CI-NEB calculations were used during this topology audit. For uniform Pd, the verified whole-network barrier was approximately 0.0231 eV during pathway screening, and a direct T-to-T linear initialisation instead produced 0.1614 eV, demonstrating that a force-converged high-symmetry band can remain on a substantially higher path.

For scaled Pd, the verified network barrier was approximately 0.0397 eV and the direct T-to-T control converged to essentially the same barrier and no lower direct route was identified. Increasing σ_{Pd} therefore retained a higher verified static barrier after the Pd topology was corrected.

S.7.6 Final Image and Force Convergence

Final numerical convergence was tested independently of pathway identification and Ni and Co were examined using 9, 13, and 17 images while uniform and scaled Pd were examined using 13, 19, and 25 images while preserving the verified network anchors. The Ni barriers were 0.151554, 0.151554, and 0.151554 eV for 9, 13, and 17 images and the corresponding Co values were 0.129377, 0.129377, and 0.129377 eV within the reported precision. For uniform Pd, the 13-, 19-, and 25-image barriers differed

by only approximately 4×10⁻⁶ eV and for scaled Pd the corresponding spread was 2.2×10⁻⁵ eV. The force-converged 13-image Ni and Co calculations at f_{tol}= 0.005 eV Å⁻¹ were retained for publication after 17-image refinement reproduced the same barriers but plateaued above the tighter force criterion. Uniform Pd satisfied the 25-image f_{tol} = 0.0025 eV Å⁻¹ criterion and scaled Pd was force-tightened using FIRE at 25 images and f_{tol} = 0.0025 eV Å⁻¹, giving 0.039822 eV.

A FIRE continuation of the Ni band collapsed onto the higher-energy T endpoint and was rejected as a saddle representation and a corresponding Co FIRE continuation did not reduce the NEB force norm and neither result was used as a publication barrier. The final publication barriers are therefore 0.151554 eV for Ni, 0.129377 eV for Co, 0.023400 eV for uniform Pd, and 0.039822 eV for scaled Pd.

The final pathway-resolved static barriers and corresponding finite-temperature Arrhenius estimates are summarised in Table S2. Static values are single-H 0 K potential-energy barriers on pathways constructed between independently verified local minima. Arrhenius Q values are effective finite-temperature barriers from the baseline 1.1 at.% H/metal MD trajectories. Static and Arrhenius values are compared by ordering and by the direction of the Pd scaling response, not by requiring numerical equality.

Table S2. Pathway-resolved 0 K static barriers and finite-temperature Arrhenius barriers.

System	Verified static pathway	Static 0 K single-H barrier (eV)	Arrhenius Q (eV)	Arrhenius 95% CI (eV)
Ni	$O \rightarrow S_{OT} \rightarrow T$	0.151554	0.2060	0.127–0.285
Co	$O \rightarrow S_{OT} \rightarrow T$	0.129377	0.1679	0.106–0.229
Pd uniform	$T_{UP} \rightarrow O^*_{UP} \rightarrow O^*_{DOWN} \rightarrow T_{DOWN}$	0.023400	0.0960	0.050–0.142
Pd scaled	$T_{UP} \rightarrow O^*_{UP} \rightarrow O^*_{DOWN} \rightarrow T_{DOWN}$	0.039822	0.1053	0.023–0.188

S.8 Concentration Sensitivity at Higher H Loading

A higher-loading Pd sensitivity test was performed to assess whether the steric-scaling direction persists beyond the baseline finite-concentration state and the test used 440 H atoms in the 8788-atom Pd supercell, corresponding to 5.0 at.% H/metal. Uniform and scaled Pd were simulated at 800, 900, 1000, and 1250 K using the same three insertion seeds 15485863, 32452843, and 49979687. Scaled Pd remained less diffusive than uniform Pd at every tested temperature and this calculation tests persistence of the scaling direction at one higher loading and does not establish concentration-independent transport kinetics.

Table S3 reports the mean diffusivities, scaled-to-uniform diffusivity ratios, and maximum swelling values for the 5.0 at.% Pd test. Replica standard deviations are represented by the error bars in Figure S1.

Table S3. Hydrogen diffusivity and stability metrics for Pd at 5.0 at.% H/metal. Diffusivities are means of three independent replicas.

Temp (K)	D uniform (m²/s)	D scaled (m²/s)	Dscaled/Duniform	Uniform max swelling (%)	Scaled max swelling (%)
800	7.074×10^{-8}	5.344×10^{-8}	0.755	0.049	0.063
900	8.746×10^{-8}	5.949×10^{-8}	0.680	0.057	0.076
1000	1.025×10^{-7}	7.141×10^{-8}	0.696	0.061	0.083
1250	1.287×10^{-7}	9.644×10^{-8}	0.749	0.093	0.120

The corresponding Arrhenius parameters and maximum swelling values for the 5.0 at.% Pd sensitivity test are summarised in Table S4.

Table S4. Arrhenius and stability summary for Pd at 5.0 at.% (H/metal convention). The fitted ***Q*** values are effective finite-temperature barriers and should be interpreted together with the diffusivity ratios in **Table S3**.

**Model**	***Q*** **(eV)**	D_0 **(m²/s)**	R^2	**Maximum swelling (%)**
Uniform Pd	0.1143	3.778×10^{-7}	0.9940	0.093
Scaled Pd	0.1161	2.777×10^{-7}	0.9813	0.120

The fitted activation-energy separation is reported for completeness and is not used as the primary evidence for the scaling effect. The higher-loading evidence is the persistent decrease in scaled-Pd diffusivity at every tested temperature together with bounded lattice swelling.

Figure S1 shows the temperature-dependent diffusivity comparison for uniform and scaled Pd at the higher H loading. The figure is included to visualise the persistent diffusivity reduction under σ scaling.

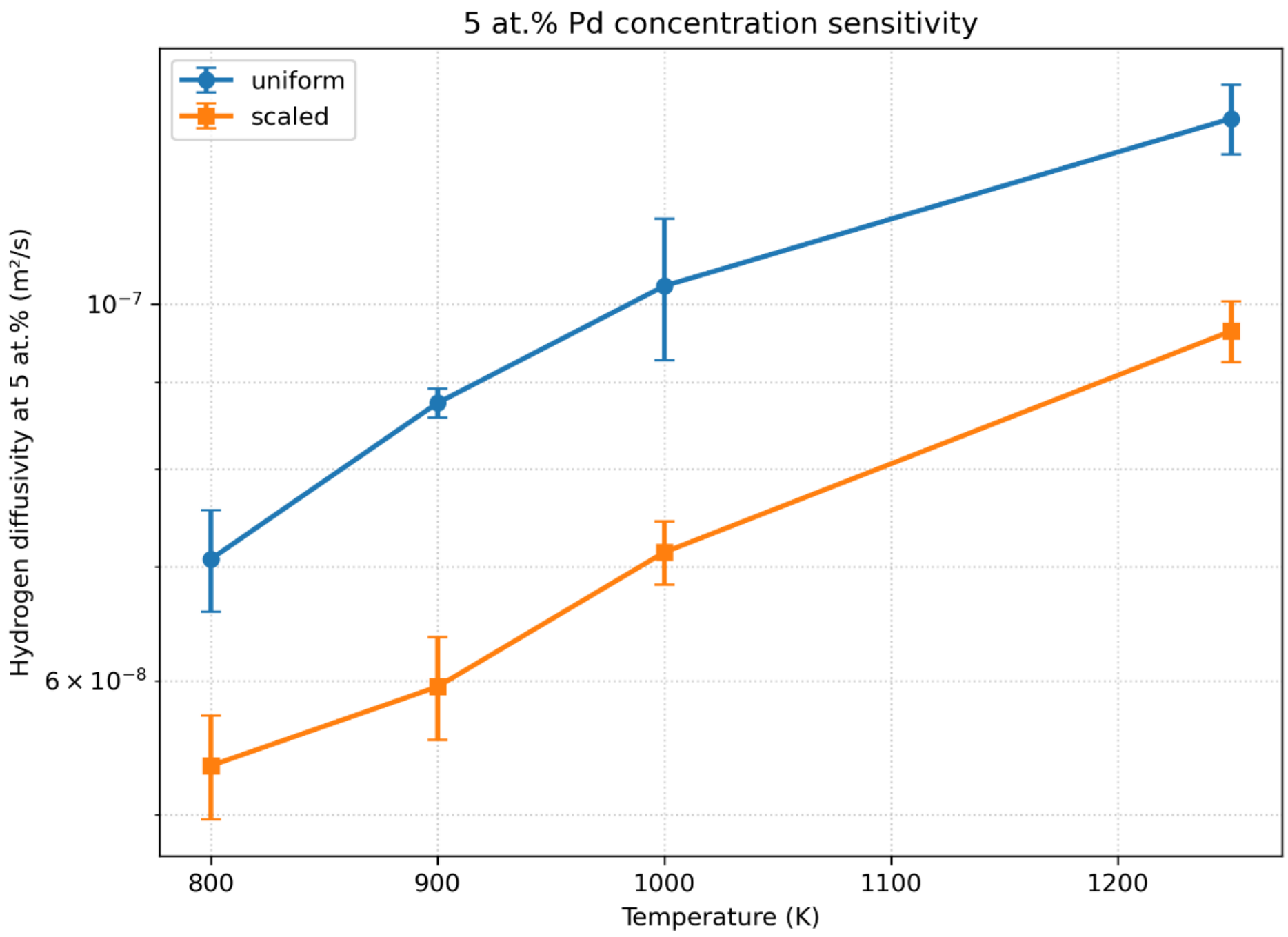

Figure S1. Hydrogen diffusivity in Pd at 5.0 at.% H/metal. Uniform and scaled parameterisations are compared at 800, 900, 1000, and 1250 K. Points show the mean of three independent replicas, and error bars show one standard deviation across replicas. Scaled Pd remains less diffusive than uniform Pd at every tested temperature.
